# Deep Learning for Object Detection, Classification and Tracking in Industry Applications

**DOI:** 10.3390/s21217349

**Published:** 2021-11-05

**Authors:** Dadong Wang, Jian-Gang Wang, Ke Xu

**Affiliations:** 1 Quantitative Imaging Research Team, Data61, Commonwealth Scientific and Industrial Research Organisation (CSIRO), Sydney, NSW 2122, Australia; 2 Institute for Infocomm Research (I2R), Agency for Science, Technology and Research (A*STAR), 1 Fusionopolis Way, #21-01, Connexis (South Tower), Singapore 138632, Singapore; jgwang@i2r.a-star.edu.sg; 3 Collaborative Innovation Center of Steel Technology, University of Science and Technology Beijing, Beijing 100083, China; xuke@ustb.edu.cn

Object detection, classification and tracking are three important computer vision techniques. They are cornerstones in the development of complex image and video analysis solutions. With the advancement of high-performance computing and storage technology, deep learning has transformed computer vision in the last decade. Numerous deep-learning-based computer vision solutions have been developed and deployed to different industry applications. For example, in manufacturing, they are used in the quality inspection of products and the visual inspection of equipment; in healthcare, computer aided diagnosis using X-ray, CT and MRI images; in agriculture, crop growth monitoring and yield estimation, animal behavior analysis and farm automation; in transportation, vehicle identification and counting, pedestrian detection and counting and road condition monitoring. Other popular applications include face and iris recognition, autonomous driving, etc.

Although the applications of object detection, classification and tracking have become ubiquitous, there are still many challenges to be addressed, such as how to curate enough good-quality training data, how to address the overfitting and underfitting issues during training, how to make the deep learning model explainable, how to quantify the uncertainty of prediction, how to deal with the data privacy issue and how to generalize a machine learning mode. The performance of deep learning models is dependent on application domains, and there is no guarantee that a machine learning model developed with training data of one domain can be used to solve problems in another domain. Therefore, domain adaptation becomes a challenging problem. For example, autonomous vehicles depend on various senor data (image, lidar, radar) to make decisions. The deep learning models developed for autonomous vehicles can detect, classify, and track objects, such as cars, pedestrians, lanes, road marks, traffic signs and lights, etc. These models need to have the domain adaptation ability to suit different weather conditions, such as rain, snow, and fog. More and more researchers are paying attention to object detection, classification and tracking under extreme conditions where the performance of the existing methods is poor.

This Special Issue aimed to provide researchers or engineers from academic and industrial backgrounds an opportunity to present their innovative solutions to address challenges in solving real-world problems using advanced machine learning and computer vision technologies. Eight papers, peer reviewed by the invited reviewers, were finally accepted and published in this issue. Xiao et al. proposed a multi-directional text detection algorithm based on improved YOLOV3 [1] to address the problem that meant the text at non-horizontal orientation could not be detectable by YOLOv3 from an image. The algorithm is capable of extending YOLOv3 from horizontal to arbitrary angles. Zhao et al. presented an end-to-end character recognition framework to recognize container characters [2]. In order to meet the high accuracy requirement in real logistics and transportation working environments, a backbone with the multi-channel split-attention module was combined with a transformer-based detection model. Video summarization research has become attractive because it facilitates large-scale video browsing and efficient video analysis. However, the computational load is high because the number of actions required to select a key frame from the video is very large. Yoon et al. [3] proposed an unsupervised video summarization method to predict importance scores by interpolating the output of a deep neural network. The piecewise linear interpolation method was utilized to mitigate the high variance problem and to generate a natural sequence of summary frames. Spark detection is important to prevent accidents caused by damaged metal. Addressing the problem that sparks could occur in aero engine chambers due to carbon deposits, lean flames or damaged metal parts, Kou et al. [4] proposed a scene-aware spark detection method in the case where aero engine anomalous spark data are lacking. Sawn timber is an important component material in furniture manufacturing, decoration, construction and other industries. In order to meet the requirements of reasonable timber use and the quality of sawn timber products, an optimized convolution neural network was proposed [5] to process sawn timber image data to identify the tree species of the sawn timber. Spatial pyramid pooling, an attention mechanism and ResNet are used to improve feature extraction and identify the sawn timber images robustly. Real time is a request for many real-world applications, e.g., autonomous vehicles, drones, and automatic production lines. One way to speed up deep learning is the use of embedded product and software, e.g., ARM-based systems. Luo et al. [6] proposed a solution to speed up a convolution neural network (CNN) with field-programmable gate arrays (FPGAs) to perform defect classification in real time. Training data curation is an important issue in deep learning research because the performance of the deep learning model is heavily dependent on the size of the training dataset. A good benchmark dataset can provide researchers with a standard to improve their own algorithms with by comparing their performance with others. Wang et al. [7] introduced a domain-specific benchmark dataset, called AgriPest, for tiny wild pest recognition and detection, providing researchers and communities with a standard large-scale dataset of wild pest images and annotations, as well as evaluation procedures. A meta-transfer learning-driven tensor-shot detector was presented [8] that decomposes the candidate scans into dual-energy tensors and employs a meta-one-shot classification backbone to recognize and localize cluttered baggage threats. The tensor-shot detector was evaluated on the publicly available SIXray and GDXray datasets.

Object detection, classification and tracking represent a collection of computer vision tasks that are far from completely solved. We hope this Special Issue will provide a reference to audiences who are interested in challenging computer vision problems in industry applications. We would be very pleased if researchers or engineers from academic or industrial backgrounds can be inspired by the contents presented in this Special Issue. 
